# Colon Conundrum: A Fascinating Case Report Unraveling the Enigmatic Tactoid Bodies

**DOI:** 10.7759/cureus.45444

**Published:** 2023-09-18

**Authors:** Kebire Gofar, Martha Yearsley, Matthew Gallardo, Hermon B Ghebrat, Subhankar Chakraborty

**Affiliations:** 1 Gastroenterology, The Ohio State University Wexner Medical Center, Columbus, USA; 2 Pathology, The Ohio State University College of Medicine, Columbus, USA; 3 Internal Medicine, The Ohio State University College of Medicine, Columbus, USA; 4 Internal Medicine, Afabet Community Hospital, Northern Red Sea Branch, Afabet, ERI; 5 Gastroenterology, The Ohio State University College of Medicine, Columbus, USA

**Keywords:** proprioception, tactoid body, s-100 protein, mechanoreceptor, intestinal obstruction

## Abstract

Tactile corpuscle-like bodies (TCLBs) are specialized mechanoreceptors found in the dermal papilla of glabrous skin. They are normally not found in the gastrointestinal (GI) mucosa. There has been an increase in incidental detection in the GI mucosa due to the widespread use of colonoscopy procedures. However, TCLB's clinical implications in the GI tract remain unknown. We present a case of a 74-year-old man who was noted to have TCLBs in the rectosigmoid mucosa following resection for iatrogenic perforation. The TCLBs were spindle-shaped, positive for S-100, and negative for CD68. We review the literature on TCLBs in the GI tract and discuss their potential function in the GI mucosa.

## Introduction

Tactile corpuscle-like bodies (TCLBs) are a type of mechanoreceptor composed of Schwann cells in the peripheral nervous system that are normally present in the skin to serve as nerve endings for pressure and vibration sensations [1]. TCLBs have also been described in the literature as Wagner-Meissner-like corpuscles, Wagner-Meissner bodies, pseudo-Meissner corpuscles, and Meissneroid corpuscles [2]. The gastrointestinal (GI) tract is normally devoid of TCLB and, if present, they are rare findings, mostly found on incidental GI endoscopies [3,4]. They are believed to be reparative neural proliferation and are deemed benign with an excellent prognosis [3]. However, there have been reports of peripheral nerve sheath tumors such as schwannomas, diffuse neurofibromas, and dermal nevi with TCLB components [1,3]. A tumor with TCLB in its entirety has also been reported in the periaortic tissue, extremities, and vulva [3]. A TCLB-associated GI tumor is unusual and almost always an incidental finding [3,4]. When present, they appear as spindle cells in the lamina propria [5]. Reports of TCLBs in the GI mucosa are exceedingly rare.

## Case presentation

A 74-year-old Caucasian male with a history of hypertension, hyperlipidemia, gastroesophageal reflux disease, transient ischemic attack, and cardiomyopathy presented with a foreign body (spray cap) in the rectum, which had been present for one day. The patient presented to the emergency department with a complaint of obstipation and discomfort. Upon questioning, he revealed that he had been suffering from erectile dysfunction for several years. In searching for natural ways to treat this problem, he read online that prostate massage could help. So he tried using an aerosol cooking spray can for that purpose. However, the cap of the can became dislodged and could not be retrieved. Due to that, he had presented to the emergency room. A flexible sigmoidoscopy was attempted but unsuccessful in removing the cap. He was thus transferred to our tertiary hospital's emergency department. A CT scan of the abdomen revealed a rectangular foreign body approximately 3.6 cm long consistent with the cap of an aerosol kitchen canister. This was noted at the level of the rectosigmoid colon. There was some thickening of the rectum at the level of the foreign body along with enhancement of the rectal mucosa and some fat stranding. There was no evidence of free air or bowel perforation. An exam under anesthesia was conducted by the trauma surgeons during which a flexible sigmoidoscopy was attempted to retrieve the cap. This was unsuccessful and in the process, the cap perforated through the rectum. He thus underwent emergency exploratory laparotomy, which revealed a perforation along the anterior wall of the proximal rectum. Segmental resection of the rectosigmoid was performed. An end colostomy was created and the rectal stump closed after confirming the absence of any leak. A gross examination of the resected specimen revealed a 1.0 cm transmural defect in the rectal wall, two flat polyps less than 1 cm in size, and a single intact diverticula. The remaining section appeared unremarkable. Pathological characteristics of the polyps, diverticula, and normal-appearing mucosa were examined.

**Figure 1:**
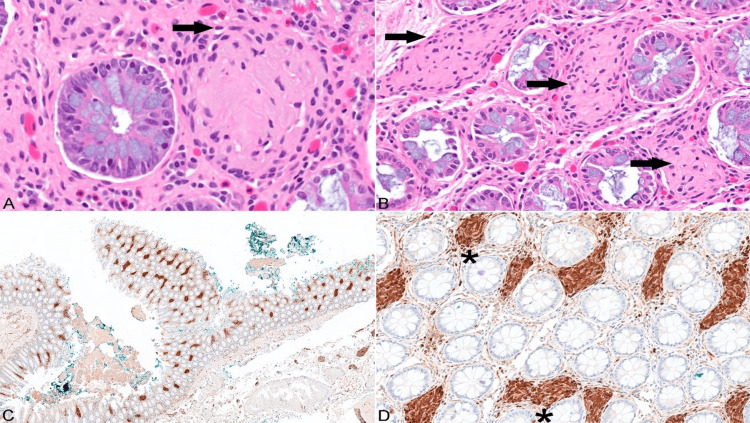
Tactile corpuscle-like bodies (TCLBs) on histologic examination of the rectosigmoid. (A) High power (400x) view of a TCLB showing spindle-shaped cells with elongated nuclei and abundant dense eosinophilic cytoplasm. (B) Lower magnification view (200x) showing numerous TCLBs in the mucosa. (C) Low power (10x) immunohistochemistry showing TCLBs in the colon lamina propria. (D) Higher magnification (200x) showing the S-100 positive TCLBs that stain brown. The asterisks denote the TCLBs highlighted by S-100.

Hematoxylin and eosin staining of the tissue revealed nuclei containing inconspicuous nucleoli with fine chromatin (Figure 1A). A densely eosinophilic cytoplasm with a layer of uniform spindle cells with indistinct cell borders within the lamina propria was noted (Figure 1B). Spindle-shaped cells diffusely positive for S-100 protein (Figures 1C, 1D) but negative for CD68, CD117, and epithelial membrane antigens were seen in the lamina propria. Immunohistochemistry was performed using an S-100 mouse monoclonal antibody (Cell Marque, Rocklin, CA). S-100 is a neural marker, positive staining supports the diagnosis of TCLBs. CD68 is a marker for macrophages. The finding of positive S-100 and negative CD-68 staining was supportive of TCLBs.

## Discussion

**Figure 2:**
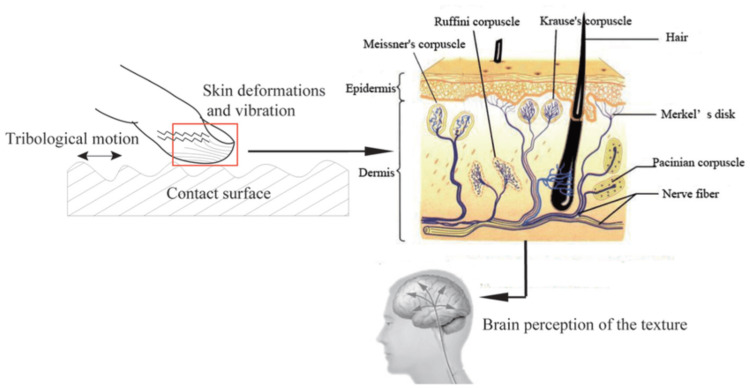
Distribution of mechanical receptors and nerve endings in the skin. Tactile receptors and nerve endings function as mechanoreceptors to detect vibrations and deformation on the skin surface. The sensation is then carried to the brain allowing us to perceive shape, hardness, moistness, and texture. Copyright/license: This figure has been adapted from an open-source article distributed under the terms and conditions of the Creative Commons Attribution Unported 3.0 (CC BY 3.0) license (https://creativecommons.org/licenses/by/3.0/). Tang W, Zhang J, Shi X, Zhang S, Liu S, Zhu H, Ge S: Investigation of mechanical responses to the tactile perception of surfaces with different textures using the finite element method. Adv Mech Eng. 2016, 8:168781401666045 [6].

Tactile perception is a result of the interaction between a stimulus pattern on the skin and the mechanical responses of receptors. Figure 2 illustrates the distribution of mechanical receptors and nerve endings throughout the skin layers. Skin receptors convert mechanical stimuli (e.g., vibration, compression, and tension) into nerve impulses that are transmitted to the brain. This tactile information travels through the nerves, spine, brain stem, and cerebral cortex, enabling the central nervous system to process it, resulting in tactile perception. Thus, humans discern object attributes like shape, hardness, moisture, and texture [6].

Microscopic Schwannian structures known as TCLBs closely mimic the superficial mechanoreceptors found in the peripheral nervous system, resembling Wagner-Meissner corpuscles. These structures have been predominantly identified in peripheral nerve sheath tumors like diffuse neurofibromas and schwannomas, as well as in cellular nevi. TCLBs are specialized mechanosensory endings originating from Schwann cells. They are situated in glabrous skin and some mucosal sites. These structures function as vital mechanoreceptors, playing a role in sensory perception.

The presence of TCLBs in the resected rectosigmoid colon represents an intriguing incidental finding in our case. TCLBs are an exceptionally rare pathologic finding in the GI tract, with only a few similar reported cases in the existing literature [3,5-7]. Their origin and role in disease pathogenesis are poorly understood.

One theory is that TCLBs are mucosal Schwann cell hamartomas that may appear endoscopically as colon polyps [8]. However, Schwann cell hamartomas are limited to the colon, while TCLBs have been reported in the esophagus, gastroesophageal junction, or stomach [4,9]. Thus, these lesions are classified as a separate entity.

Histologically, TCLBs mimic mucosal granuloma and amyloid depositions [3]. However, unlike amyloid deposits, TCLBs do not stain with Congo red [3,10]. In contrast to mucosal granulomas, TCLBs are generally positive for S-100 but negative for CD68 [3,10].

The significance of TCLB in the context of colorectal foreign bodies is unknown. Though there is no established causal relationship between these entities. One possibility is that the presence of a foreign body in the rectum may have led to mucosal irritation or inflammation, potentially triggering the formation of these structures. Alternatively, TCLBs could represent a separate incidental finding unrelated to the rectal foreign body. Additional studies may help identify associations or patterns between TCLBs and rectal foreign bodies.

This report underscores the increasing incidence of incidentally discovered TCLBs in various clinical presentations. While our patient had an uneventful recovery, and we believe the condition was an isolated finding, potential associated lesions or syndromes should not be overlooked. Further studies are needed to further understand the etiology, clinical implications, and long-term outcomes of TCLB in the GI mucosa. Immunohistochemically, this condition should not be confused with other conditions with similar histological characteristics.

## Conclusions

TCLBs are a rare finding in the mucosa of the GI tract. We hypothesize that the TCLBs in our patient were either a hamartoma or resulted from trauma from rectal perforation. Their role in the pathogenesis of perforation in the patient and in the GI tract remains to be elucidated.
